# Description of the male and the larva of *Ixodes collaris* Hornok, 2016 with drawings of all stages

**DOI:** 10.1186/s13071-019-3365-3

**Published:** 2019-03-26

**Authors:** Sándor Hornok, Dávid Murányi, Jenő Kontschán, Vuong Tan Tu

**Affiliations:** 1 Department of Parasitology and Zoology, University of Veterinary Medicine, Budapest, Hungary; 2 Plant Protection Institute, Centre for Agricultural Research, Hungarian Academy of Sciences, Budapest, Hungary; 3 Institute of Ecology and Biological Resources, Vietnam Academy of Science and Technology, Hanoi, Vietnam; 4 Graduate University of Science and Technology, Vietnam Academy of Science and Technology, Hanoi, Vietnam

**Keywords:** *Ixodes collaris*, Bat tick, Cave, Male, Larva, Description

## Abstract

**Background:**

*Ixodes collaris* Hornok, 2016 is a recently discovered tick species associated with bats in Asia. This study provides the description of the male and the larva, as well as high quality drawings of all stages.

**Methods:**

Ticks were collected from cave walls and bats in Phia Oac (Vietnam). DNA was extracted from one individual of each stage/sex, while another was morphometrically analysed. Based on two genetic markers, all ticks were identified as *I. collaris*.

**Results:**

The male of *I. collaris* has long legs (i.e. the length of Haller’s organ exceeds the maximum diameter of tarsus I), unlike the male of *I. simplex* Neumann, 1906, but similarly to males of *I. vespertilionis* Koch, 1844 and *I. ariadnae* Hornok, 2014. The lateral and medial edges of the palpi of male *I. collaris* are both convexly curved, unlike in *I. ariadnae* and *I. simplex*, but similarly to *I. vespertilionis*. The male of *I. collaris* has long palpal setae (up to 210 µm), unlike the males of *I. ariadnae* (30–100 µm) and *I. simplex* (20–80 µm), but similarly to *I. vespertilionis* (100–200 µm). Males of *I. collaris* have sparse distribution of long palpal setae (*vs* dense in *I. vespertilionis*) and posteriorly diverging, sclerotized trapezoid ridge dorsally on the basis capituli (posteriorly convergent, U-shaped and less evident in *I. vespertilionis*). The larva of *I. collaris* has long legs (unlike the larva of *I. simplex*, but similarly to *I. vespertilionis* and *I. ariadnae*), elongated club-shaped palpi (240 × 70 *vs* 200 × 90 µm in *I. ariadnae*, 200 × 70 µm in *I. vespertilionis*; and 140 × 60 µm in *I. simplex*:), pentagonal scutum, which is longer than broad (different from *I. ariadnae* and *I. simplex*, but similar to that of *I. vespertilionis*). The larva of *I. collaris* has strongly concave caudolateral margin of ventral basis with perpendicular angle (*vs* slightly concave, with obtuse angle in *I. vespertilionis*) and a prominent, dark sclerotized edge, “collar” (absent in *I. vespertilionis*).

**Conclusion:**

Several features allow to distinguish the male and the larva of *I. collaris* morphologically from those of other bat-associated ixodid tick species.

## Background

In a recent Eurasian survey on ixodid ticks infesting bats, high degree of mitochondrial gene heterogeneity of *Ixodes vespertilionis* Koch, 1844 was reported, postulating that it is actually a species complex, within which hitherto unknown or formerly not distinguished bat tick species might exist [1]. Accordingly, *I. collaris* Hornok, 2016, has been described, based on material from the intermediate horseshoe bats (*Rhinolophus affinis*) in Vietnam. However, the description of this new species was based only on the female and the nymph [2], because at that time male bat ticks were not seen on cave walls and larvae could not be collected from the only known host species.

This study provides the description of the male and the larva of *I. collaris*, in order to complete the description of the species. In addition, because detailed drawings of the female and the nymph of *I. collaris* have not been available, high quality drawings of its all stages are also provided here.

## Methods

### Sample collection

*Ixodes* sp. ticks (two individuals per stage or sex) were collected at a cave located in the buffer zone of Phia Oac, Phia Den National Park (22.563611N, 105.874167E), Cao Bang Province, Vietnam (i.e. the type-locality of *I. collaris*) on June 1, 2016 and November 17, 2017. Male ticks were removed from cave walls, whereas females, nymphs and larvae were collected from *R. affinis*. All specimens were stored in 96% ethanol. After microscopical evaluation of their conspecificity, DNA was extracted from one individual of each stage/sex, while the other was morphometrically analysed and described. Specimens of *I. vespertilionis* (used for comparison) were collected in Leány cave, Hungary.

### Sample analyses

From the DNA extracts, molecular analyses of two mitochondrial markers were performed as reported [1] by two PCRs: the first amplifying an approximately 710 bp long fragment of the cytochrome *c* oxidase subunit 1 (*cox*1) gene with the primers HCO2198 (5′-TAA ACT TCA GGG TGA CCA AAA AAT CA-3′) and LCO1490 (5′-GGT CAA CAA ATC ATA AAG ATA TTG G-3′), and the second targeting an approximately 460 bp fragment of the *16S* rRNA gene of Ixodidae with the primers 16S + 1 (5′-CTG CTC AAT GAT TTT TTA AAT TGC TGT GG-3′) and 16S-1 (5′-CCG GTC TGA ACT CAG ATC AAG T-3′). PCR products were visualized in a 1.5% agarose gel. Purification and sequencing was done by Biomi Inc. (Gödöllő, Hungary). Sequences were compared to those already deposited in GenBank by nucleotide BLASTN program (https://blast.ncbi.nlm.nih.gov).

Pictures and measurements were made with a VHX-5000 (Keyence Co., Osaka, Japan) digital microscope.

## Results

Based on the amplified fragment of their *cox*1 and *16S* rRNA genes, all ticks described here were identified as *I. collaris*, having 100% sequence identity to formerly reported female and nymph specimens of this tick species (*cox*1 gene: KR902756, *16S* rRNA gene: KR902771). The newly generated sequences were submitted to the GenBank database under the accession numbers MK450318-MK450319 (*cox*1) and MK450320-MK450321 (*16S* rRNA). The measurements in the descriptions below are provided in millimetres.

## ***Ixodes collaris*** Hornok, 2016

### Description

**Fig. 1:**
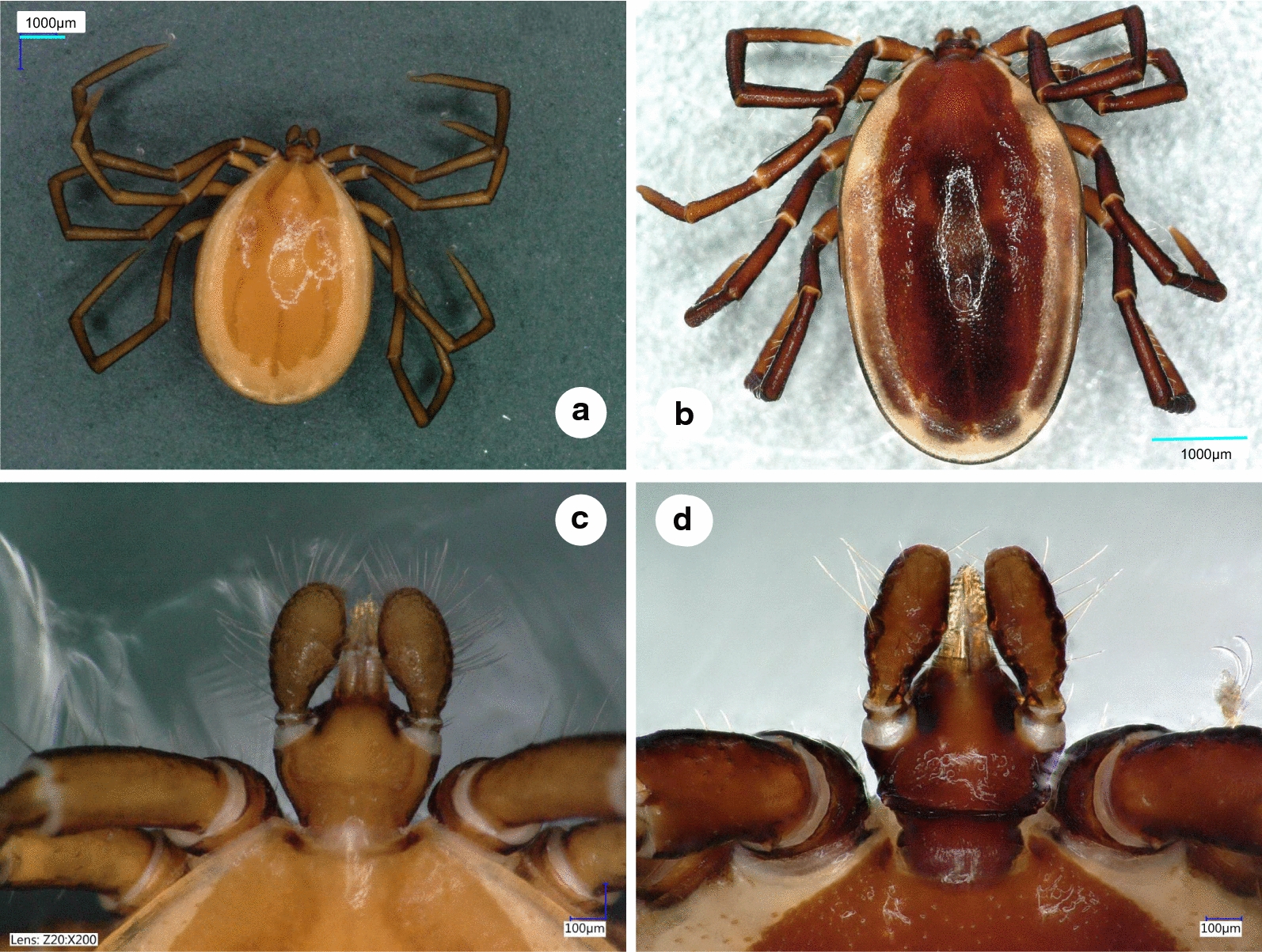
Dorsal views of male *Ixodes vespertilionis* (**a**, habitus; **c**, gnathosoma) and *I. collaris* (**b**, habitus; **d**, gnathosoma)

**Fig. 2:**
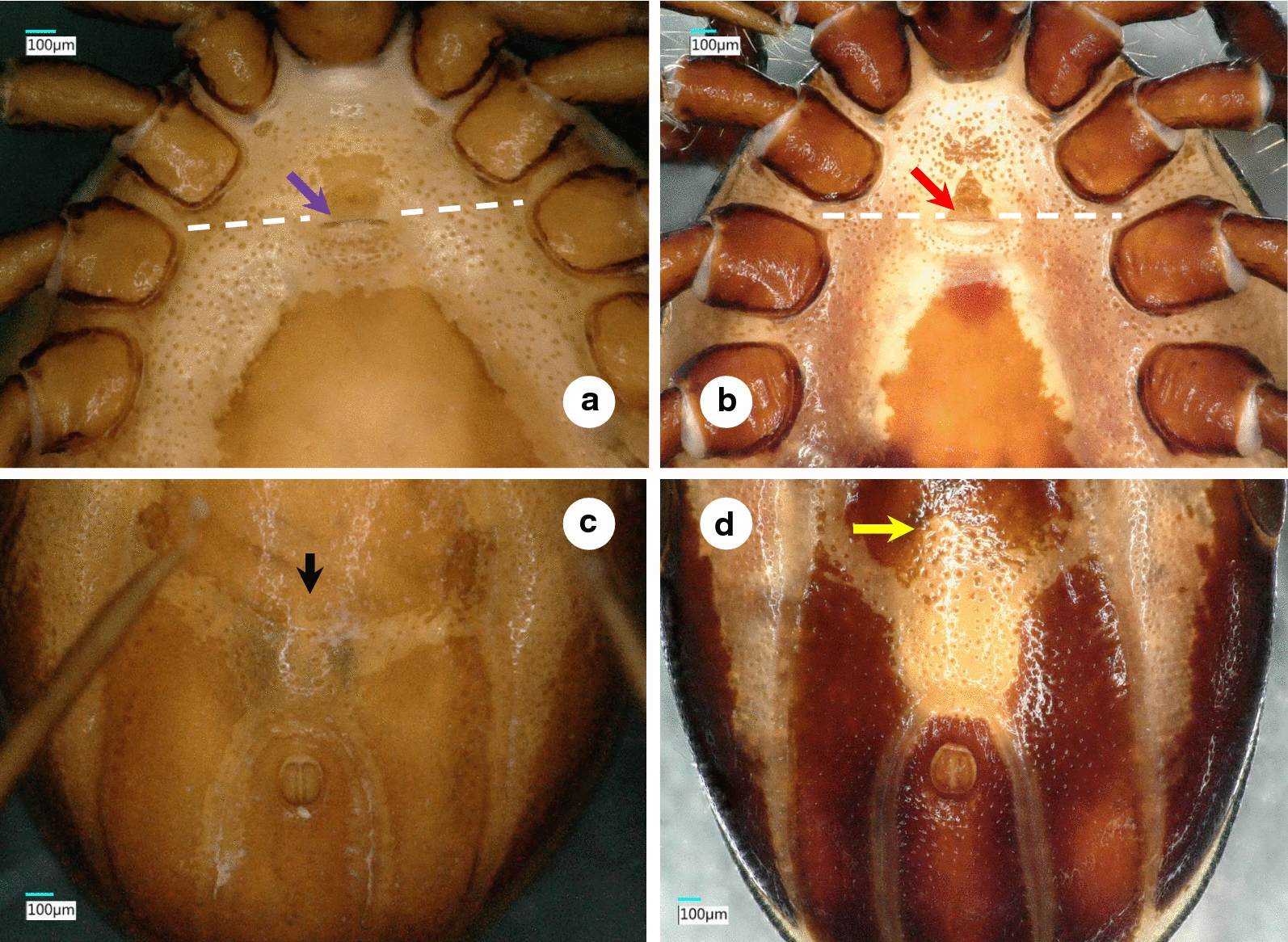
Ventral views of male *Ixodes vespertilionis* (**a**, anterior part of the idiosoma; **c**, posterior part of the idiosoma) and *I. collaris* (**b**, anterior part of the idiosoma; **d**, posterior part of the idiosoma). The white dashed line indicates the second intercoxal space. The purple and red arrows show the genital aperture. The black and yellow arrows point to differences in the anterior part of the ivory coloration

**Fig. 3:**
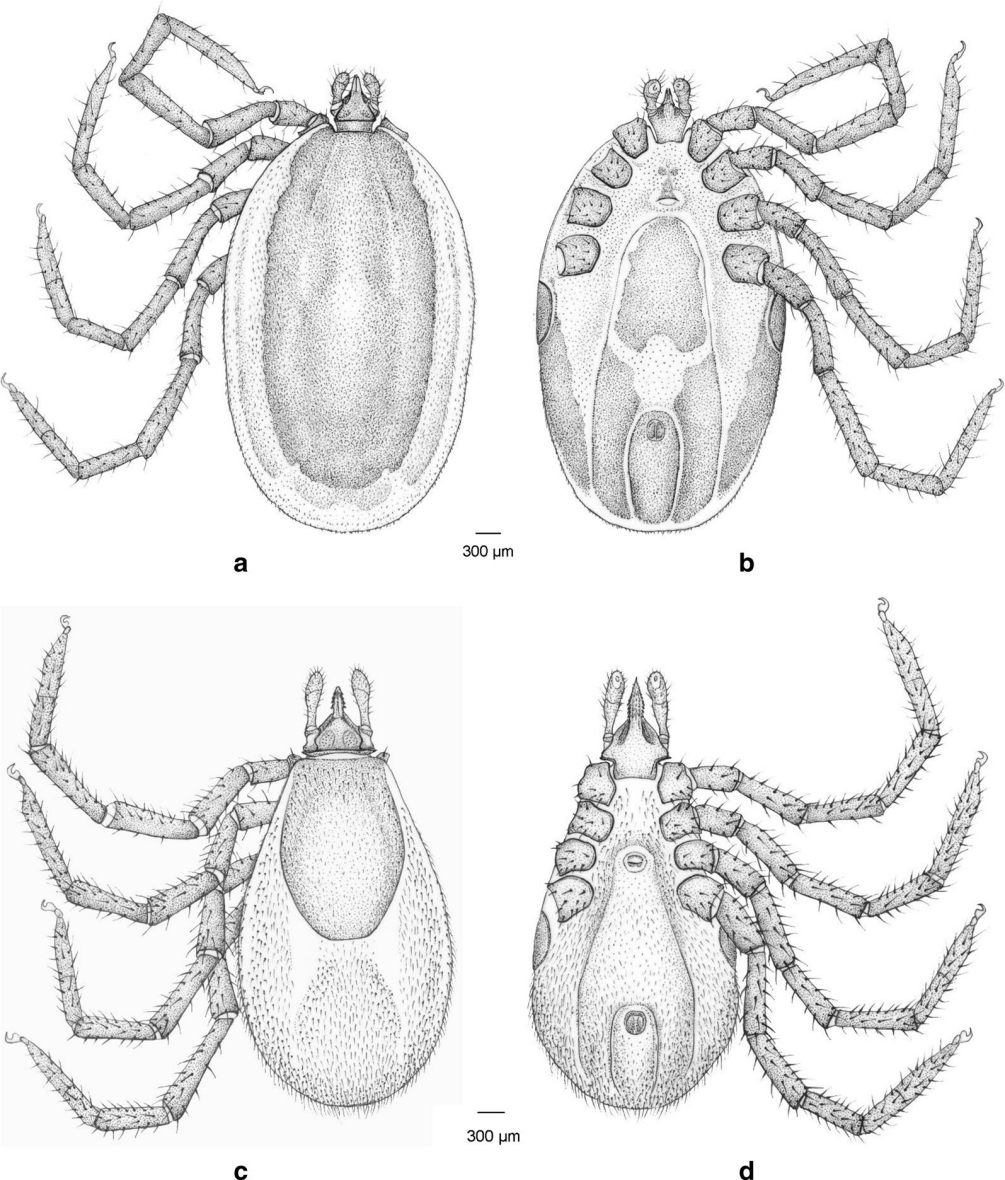
Drawings of *Ixodes collaris* male (**a**, dorsal view; **b**, ventral view) and female (**c**, dorsal view; **d**, ventral view)

***Male.*** [See Figs. 1–3.] Length of idiosoma (from half point between scapular apices to posterior margin) 4.49, breadth 2.78, ratio of idiosomal length/breadth 1.61. Conscutum centrally dark brown, laterally lightly colored, elongated, elliptical, broadest slightly anteriorly to its mid-length (Fig. 1b). Length of scutum 4.2, breadth 2.45, ratio length/breadth 1.71. Cervical grooves anteriorly well-defined, paramedian grooves posteriorly shallow; scattered punctuations present. Genital aperture at level of second intercoxal space, surrounded by ivory color and scattered punctuations (Fig. 2b). Spiracular plates slightly oval (length/breadth ratio 1.23), with eccentric opening. Ivory coloration anterior to anus oval, elongated. Anal plate oval, elongated, broadest at mid-length. Adanal plates 1.5 times longer than anal plate. Median plate anteriorly rounded, longer than broad (Figs. 2b, d, 3b).

Gnathosoma: length from palpal apices to posterior margin of basis 0.78, width of basis dorsally 0.48, ratio of length to width 1.63. Basis capituli dorsally broadest at base of palpi, with laterally and posteriorly elevated, backwardly diverging (trapezoid), dark, sclerotized ridge (Fig. 1d). Basis capituli ventrally trapezoidal, posteriorly tapering (Fig. 2b). Palpi relatively short, length (dorsally) 0.53, breadth 0.21, ratio length/breadth 2.52. Palpal segment I. 0.04, II-III (with indistinct separation) 0.48 long. Segments II-III curved both medially and laterally, with uneven surface (Fig. 1d). Palpal setae long anteriorly, showing sparse distribution laterally (less than 10 in number), on segments II-III up to 0.21 long (i.e. equal to width of palpi (Fig. 1d). Hypostome conical, length 0.26, breadth at basis 0.15, ratio length/breadth 1.73. Teeth poorly defined, with dental formula 3/3.

Legs long. Hallerʼs organ elongated, longer than maximum breadth (diameter) of tarsus I (length: 1.37). Coxae medially rounded, without spines or spurs, but with short (0.07) setae (Figs. 2b, 3b).

**Fig. 4:**
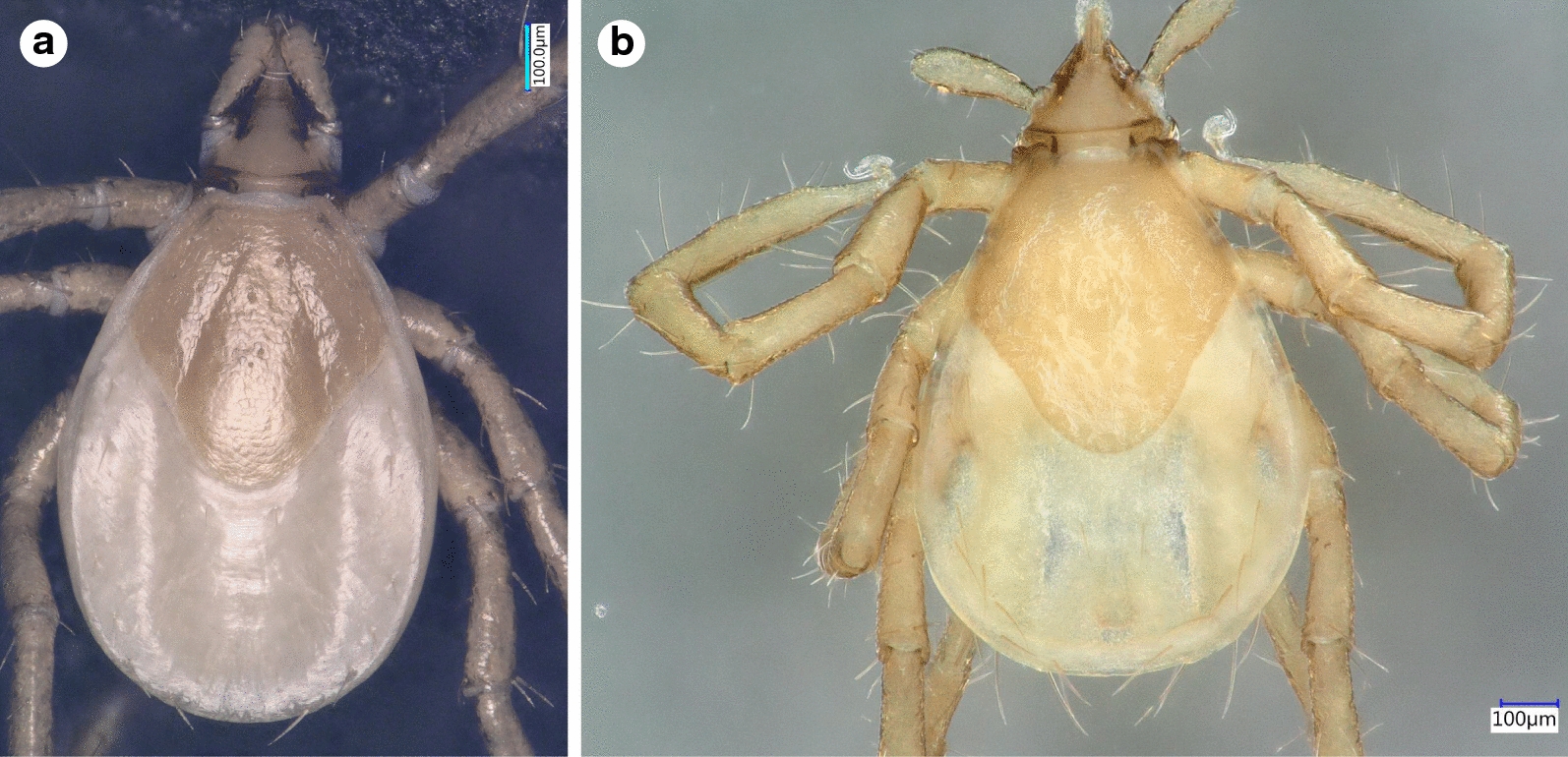
Dorsal view of *Ixodes vespertilionis* (**a**) and *I. collaris* (**b**) larvae

**Fig. 5:**
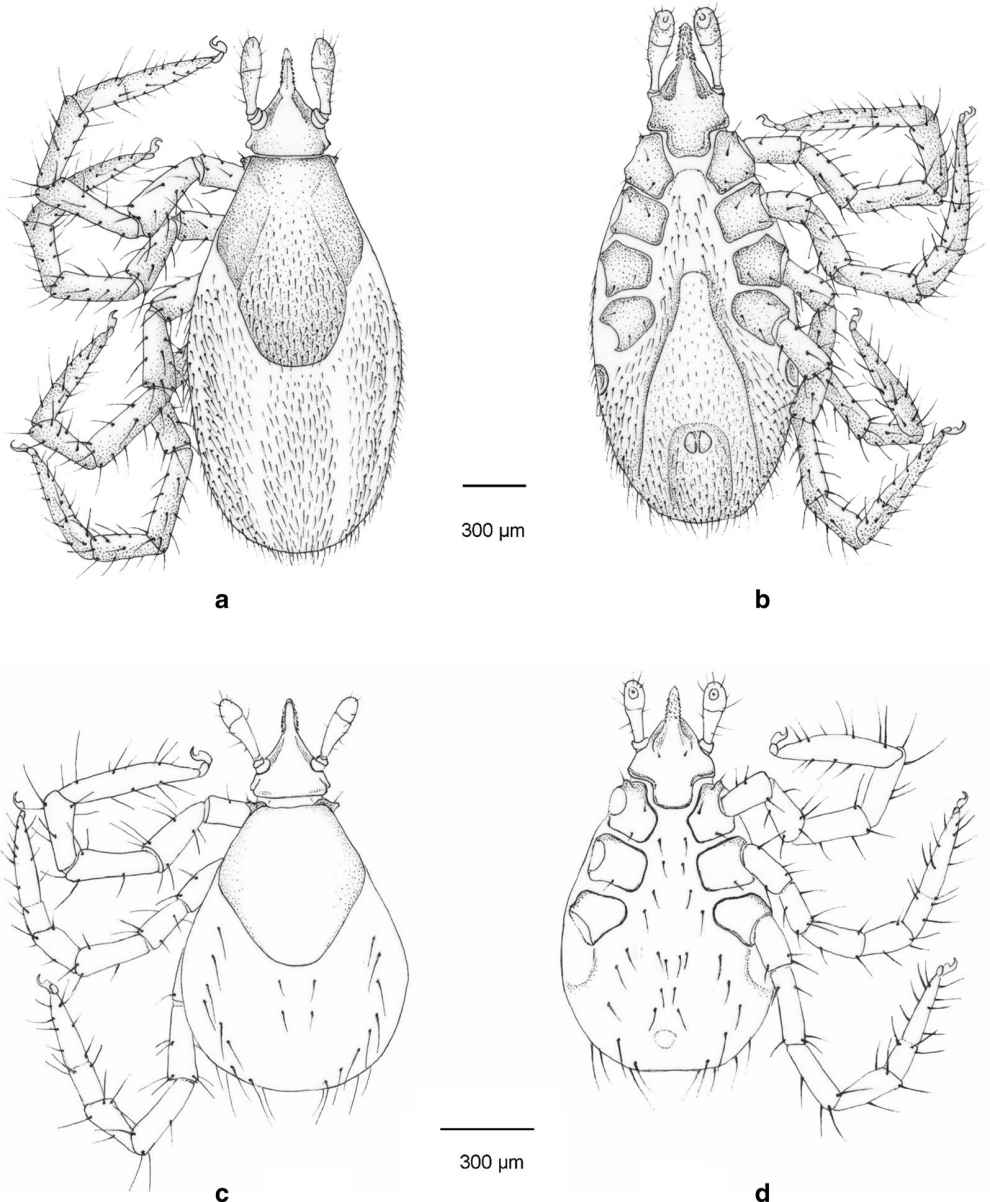
Drawings of *Ixodes collaris* nymph (**a**, dorsal view; **b**, ventral view) and larva (**c**, dorsal view; **d**, ventral view)

**Fig. 6:**
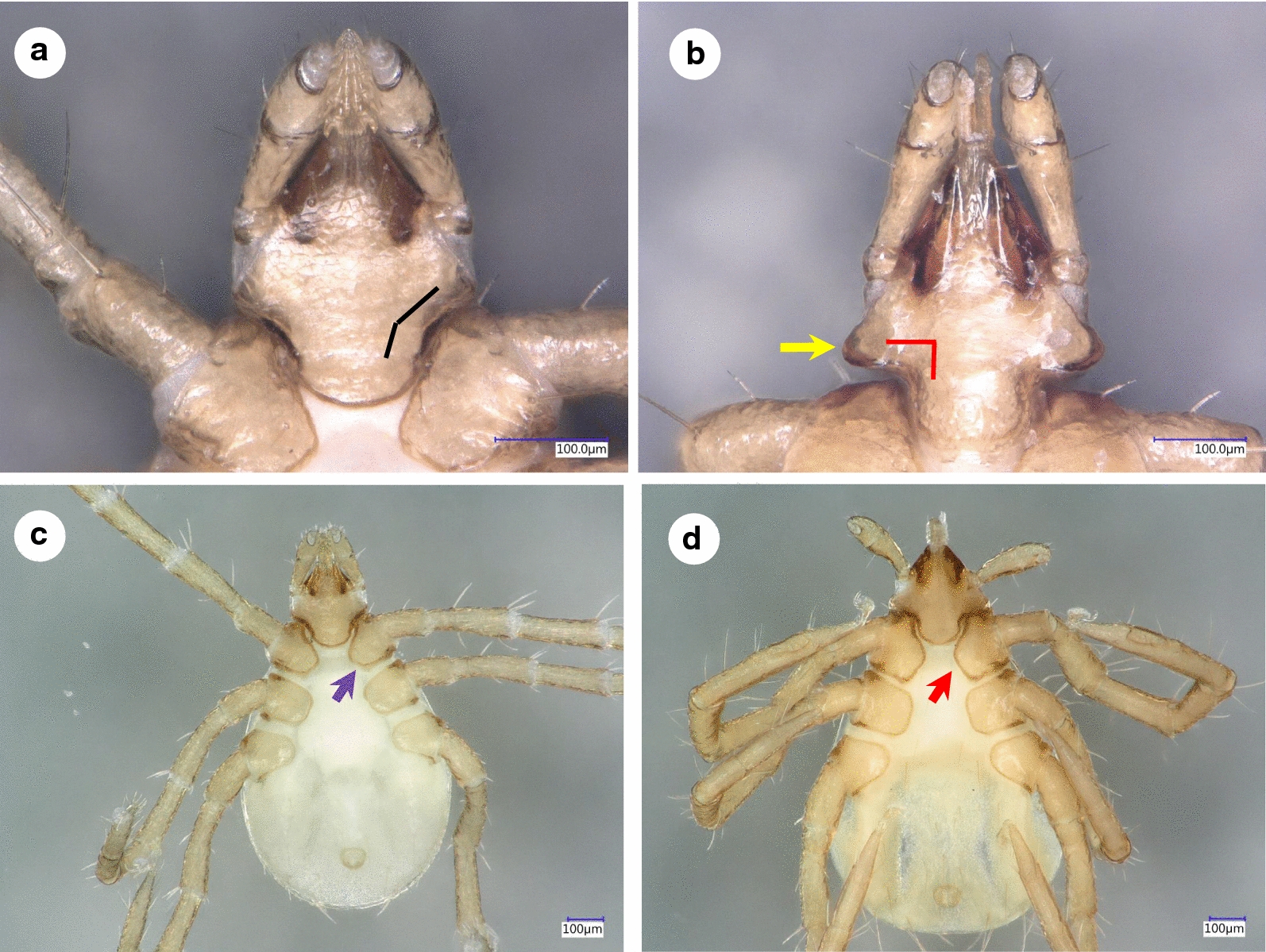
Ventral views of larvae of *Ixodes vespertilionis* (**a**, gnathosoma; **c**, habitus) and *I. collaris* (**b**, gnathosoma; **d**, habitus). The black and red broken lines illustrate the angle of the caudolateral edge of the gnathosoma. The yellow arrow marks the “collar”. The purple and red arrows mark the medial edge of coxa I

***Larva.*** [See Figs. 4–6.] Length of idiosoma 0.96, breadth 0.76, ratio idiosomal length/breadth 1.26. Scutum pentagonal, posteriorly rounded, broadest slightly anteriorly to its mid-length (Fig. 4b), with rough surface. Length of scutum 0.52, breadth 0.49, ratio length/breadth 1.06. Cervical grooves long, ending at deepest point of concavely curved posterolateral margin of scutum (Fig. 4b). Scutal setae absent. Alloscutal setae longest caudally; central dorsal setae (Cd1: 0.045, Cd2: 0.047) shorter than marginal dorsal setae (Md1-Md8: 0.088, 0.096, 0.105, 0.112, 0.125, 0.108, 0.133 and 0.148, respectively). Sternal setae ventrally (St1: 0.056, St2: 0.051; St3: 0.062) shorter than premarginal setae (Pm1-Pm4: 0.072, 0.075, 0.076 and 0.071, respectively) and preanal setae (Pa1: 0.064; Pa2: 0.078); marginal ventral setae longest (Mv1: 0.092; Mv2: 0.118; Mv3: 0.128) (Fig. 5c, d).

Gnathosoma: length from palpal apices to posterior margin of basis 0.34, width of basis capituli dorsally 0.25, ratio of length to width 1.36. Basis capituli dorsally triangular, with straight posterior margin. Ventral basis with 2 pairs of short (0.015) posthypostomal setae. Strongly concave caudolateral margin of ventral basis with perpendicular angle and dark sclerotized edge (“collar”) at and behind maximum width of basis (Fig. 6b). Posterior margin of ventral basis rounded (Fig. 6b). Palpi elongated, club-shaped, curved both medially and laterally. Palpal length dorsally 0.24, breadth 0.07, ratio length/breadth 3.4. Segments I-III measure 0.03, 0.13 and 0.08, respectively. Palpal setae longest (0.06) near junction of segments II-III. Hypostome conical, short (0.11), with dental formula 2/2.

Legs long. Hallerʼs organ elongated, longer than maximum breadth (diameter) of tarsus I (length: 0.48) (Figs. 4b, 5c, d, 6d,). Coxae medially rounded, without spines or spurs. Medial margins of coxae I-II straight, nearly equal in length; those of coxae III rounded (Fig. 6d). Coxae I-II with short (0.04) setae (2 and 1, respectively: Fig. 5d).

### Differential diagnosis

Prior to its discovery, *I. collaris* may have been misidentified as *I. vespertilionis* in Southeast Asia (e.g. in [3]). Therefore, the differential diagnosis (see Figs. 1, 2, 4 and 6) focuses on the latter species, as redescribed in [4] and [5]. Apart from data taken from the latter source on larvae of *I. simplex* Neumann, 1906, the redescription of the male of *I. simplex* was also taken into account [6], together with measurements (including those of *I. ariadnae* Hornok, 2014) in [7].

The male of *I. collaris* has long legs (i.e. the length of Haller’s organ exceeds the maximum diameter of tarsus I), unlike the male of *I. simplex*. The palpi of *I. collaris* male have convexly curved lateral and medial edges, unlike the males of *I. ariadnae* (where the lateral edge is straight) and *I. simplex* (where the lateral edge is bent at an angle). The palpal setae are long (up to 210 µm), unlike in the males of *I. ariadnae* (30–100 µm) and *I. simplex* (20–80 µm). The most important features to distinguish males of *I. collaris* from those of *I. vespertilionis* are on the gnathosoma: (i) more elongated palpi (length to breadth ratio 2.52 *vs* 2.0–2.1) (Fig. 1d *vs* 1c); (ii) the sparse distribution and low number (< 10) of long anterior and lateral palpal setae, which show dense distribution and higher number (> 20) in *I. vespertilionis* (Fig. 1c); and (iii) the posteriorly diverging (trapezoid), sclerotized ridge dorsally on the basis capituli, which is much less conspicuous and posteriorly converging (U-shaped) in *I. vespertilionis* (Fig. 1c). In addition, (iv) the idiosoma of male *I. collaris* is more elongated than the idiosoma of male *I. vespertilionis* (length to breadth ratio 1.61 *vs* 1.35, Fig. 1b *vs* 1a) and (v) ventrally the ivory coloration anterior to the anus is oval, elongated in *I. collaris*, whereas this is less apparent in *I. vespertilionis* (Fig. 2c).

The larva of *I. collaris* has long legs, unlike the larva of *I. simplex* (in which the legs are short, i.e. the length of Haller’s organ does not exceed the maximum diameter of tarsus I). The palpi of *I. collaris* larva are elongated, club-shaped, with a length to breadth ratio above three (length × width: 240 × 70 µm), unlike in *I. vespertilionis* (Fig. 6a: club shaped, but shorter, 200 × 70 µm), *I. ariadnae* (shorter and broader, 200 × 90 µm, laterally straight) and *I. simplex* (shorter and narrower, 140 × 60 µm). The scutum of *I. collaris* larva is longer than broad, pentagonal, with long cervical grooves, ending at the deepest point of the concavely curved posterolateral margin of scutum (unlike in case of *I. ariadnae* and *I. simplex*). The most important features to distinguish the larva of *I. collaris* from that of *I. vespertilionis* can be observed ventrally on the basis capituli: (i) its caudolateral edge is strongly concave in case of *I. collaris*, showing perpendicular angle at the concavity, whereas it is only slightly concave, with an obtuse angle, in *I. vespertilionis* (Fig. 6a); and (ii) at/behind the maximum breadth of ventral basis, the presence of a conspicuous, long, dark sclerotized edge (“collar”) also makes *I. collaris* larvae different from *I. vespertilionis* larvae, where this structure is less apparent (Fig. 6b *vs* 6a). In addition, (iii) caudal setae on the idiosoma are considerably longer than 100 µm in *I. collaris* larvae, unlike in *I. vespertilionis* larvae (≤ 100 µm, see Figs. 4a and 6c); and (iv) the medial edge of coxa I is much shorter than that of coxa II in *I. vespertilionis* larvae (Fig. 6c), as contrasted to *I. collaris* larvae.

## Discussion

With the morphological characters, high resolution pictures and drawings provided in the present study, descriptions of all stages of *I. collaris* are now complete. Adding to already reported genetic differences between *I. vespertilionis*, *I. collaris*, *I. ariadnae* and *I. simplex* [1], these ixodid bat tick species show different morphology in all stages, as shown formerly for females and nymphs [2] and here for males and larvae.

In summary, short legs distinguish all stages of *I. simplex* from *I. collaris*. *Ixodes ariadnae* has laterally straight and short palpi in all stages, unlike *I. collaris*. The male of *I. vespertilionis* has dense long palpal setae (sparse in *I. collaris*), whereas its females, nymphs and larvae lack the ventrolateral collar of gnathosoma (characteristic of *I. collaris*).

## Conclusions

Based on the descriptions above, several features allow to distinguish the male and the larva of *I. collaris* morphologically from those of other bat-associated ixodid tick species.
